# Isolation and full-length genome analysis of mosquito-borne Manzanilla virus from Yunnan Province, China

**DOI:** 10.1186/s13104-015-1198-5

**Published:** 2015-06-23

**Authors:** Yun Feng, Shi-Hong Fu, Wei-Hong Yang, Yu-Zhen Zhang, Biao He, Chang-Chun Tu, Guo-Dong Liang, Hai-Lin Zhang

**Affiliations:** Yunnan Institute of Endemic Diseases Control and Prevention, Yunnan Provincial Key Laboratory for Zoonosis Control and Prevention, Dali, 671000 People’s Republic of China; State Key Laboratory for Infectious Disease Prevention and Control, National Institute for Viral Disease Control and Prevention, Chinese Center for Disease Control and Prevention, Beijing, 102206 People’s Republic of China; Institute of Military Veterinary, Academy of Military Medical Sciences, Changchun, Jilin 130062 People’s Republic of China

**Keywords:** Manzanilla virus, Oya virus, Orthobunyavirus, Phylogenetics

## Abstract

**Background:**

There have been four strains on Manzanilla virus (MANV) identified to date. Here, we identify a novel MANV strain (DHL10M107) isolated from *Culex tritaeniorhynchus* Giles mosquitoes from Ruili city, Dehong prefecture, Yunnan Province, in the People’s Republic of China.

**Results:**

The DHL10M107 L, M and S genes were sequenced at the nucleotide and deduced amino acid levels. The L, M and S gene sequences of DHL10M107 clustered with the MANV strains VN04-2108, TRVL3587, SA An 4165, and AV 782. DHL10M107 was most closely related to VN04-2108. Nucleotide homology ranged between 96 and 99% between DHL10M107 and VN04-2108. In terms of amino acid homology, all of the amino acid differences were in the L (96.3% homologous) and M (97.7% homologous) fragments.

**Conclusions:**

DHL10M107 is likely a MANV isolated from mosquitos in the Yunnan Province. This is the first reported isolation of MANV in mainland China.

**Electronic supplementary material:**

The online version of this article (doi:10.1186/s13104-015-1198-5) contains supplementary material, which is available to authorized users.

## Findings

Manzanilla virus (MANV) belongs to the Simbu serogroup of the genus *Orthobunyavirus* of family *Bunyaviridae* [1–3]. MANV is a single-stranded negative sense RNA virus that contains three RNA fragments: large (L), medium (M) and small (S). Four strains of MNV have been isolated from to date. Anderson et al. were the first group to isolate MANV (TRVL3587) [1] from the blood of a Howler monkey (*Alouatta seniculus insularis*) in Trinidad in 1954. More recently, in 2004 Bryant et al. isolated a MANV strain identified as the Cat Que virus (VN04-2108) from mosquitoes (*Culex* sp.) in Vietnam [2]. In 2014, Ladner et al. [3] reclassified two viruses as MANV strains, the Ingwavuma virus (SA An 4165) isolated from a South African spectacled weaver (*Hyphanturgus ocularis*) in 1959 [4], and the Mermet virus (AV 782) isolated from a North American purple martin (*Progne subis*) in the United States in 1964 [5].

Yunnan Province is located in southwest China, adjacent to Myanmar, Laos and Vietnam. Ecologically, Yunnan Province supports the distribution of vector mosquitoes and the transmission of mosquito-borne viruses [6, 7]. The Japanese encephalitis virus (Genus *Flavivirus*, Family *Flaviviridae*), Dengue virus (Genus *Flavivirus*, Family *Flaviviridae*), Chikungunya virus (Genus *Alphavirus*, Family *Togaviridae*), Sindbis virus (Genus *Alphavirus*, Family *Togaviridae*), Getah virus (Genus *Alphavirus*, Family *Togaviridae*), Batai virus (BATV) (Genus *Orthobunyavirus*, Family *Bunyaviridae*), Banna virus (Genus *Seadornavirus*, Family *Reoviridae*), Yunnan orbivirus (Genus *Obivirus*, Family *Reoviridae*), and several other viruses have been isolated from human patients and from mosquitoes collected in Yunnan Province [8–16]. This study aimed to further investigate the distribution of mosquito-borne viruses in Yunnan Province.

In August 2010, mosquitoes were collected using the Kongfu Xiaoshuai trap (Wuhan Jixing Environmental Protection, Scientific, and Technological LLC) from cattle barns in the suburb of Ruili city, Dehong prefecture, Yunnan Province, People’s Republic of China. A total of 425 mosquitoes representing 10 species in four genera were collected. Of these, 318 (74.82%) were *Culex tritaeniorhynchus* Giles and the remaining 107 were from the other nine species (25.18%). The mosquito samples were sorted by species into 15 pools and ground together. The supernatant was used to inoculate Baby hamster kidney (BHK-21) cells to isolate any viruses present as previously described [15]. One of the pools containing *C. tritaeniorhynchus* (DHL10M107) caused significant cytopathic effects (CPE) in BHK-21 cells, characterized by the cells shrinking, rounding, and eventually floating.

The viral RNA was extracted from the culture supernatant of the DHL10M107 isolate, using the QIAamp viral RNA mini kit (Qiagen, Valencia, CA, USA) per the manufacturer’s instructions. cDNA was prepared with Ready-To-Go You-Prime First-Strand Beads™ (American Amersham Pharmacia Biotech, Piscataway, NJ, USA), and amplified with MANV specific primers (see Additional file 1: Table s1). The reactions contained 1 µL cDNA template, 10 µL 2 × GCI Buffer, 0.5 µL 2.5 mM dNTP, 0.4 µL each of the upstream and downstream primers (10 mM), 0.4 µL ExTaq enzyme (5 U/µL), and 7.3 µL deionized water. The reaction conditions were: pre-denaturing at 95°C for 3 min, followed by 32 cycles of 95°C for 30 s, 58°C for 40 s, and 72°C for 1–2 min, and extension at 72°C for 10 min. The amplification was assessed using 2 µL of the product for 1% agarose gel electrophoresis. The whole genome sequence of the DHL10M107 strain was obtained (Beijing Liuhe Genomics Technology Co., Ltd. Shenzhen Branch). The L, M and S sequences of DHL10M107 strain were submitted to GenBank under accession nos. KP016012-KP016014. The sequence fragments were spliced, edited and corrected using SeqMan (DNAstar software package) and the phylogenetic analysis and nucleotide sequence analysis were performed using ClustalX (version 1.8), MEGA5.0, DNAStar alignment package, and MegAlign software.

**Figure 1:**
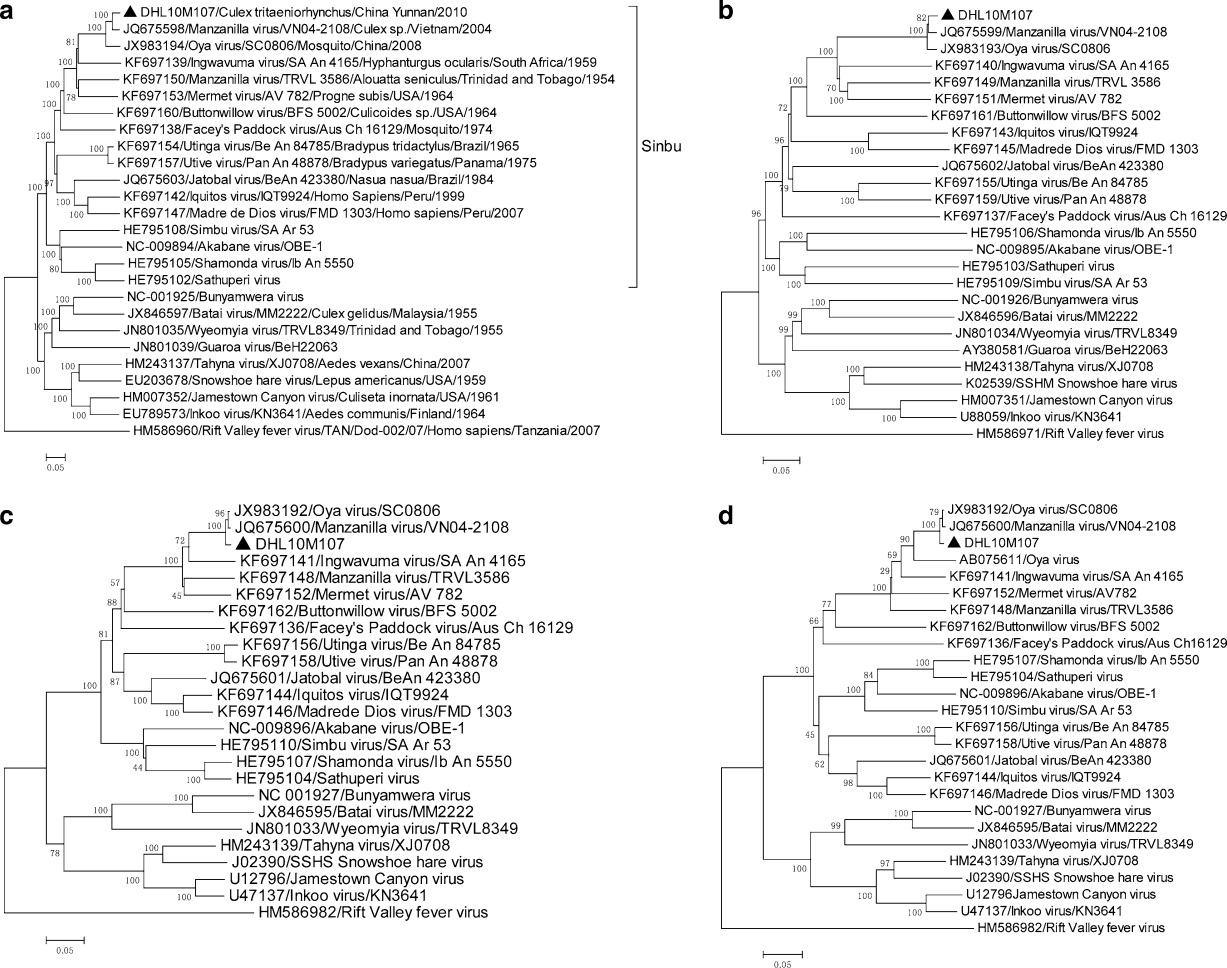
Phylogenetic trees based on the nucleotide sequences of DHL10M107 strains isolated from Yunnan Province, China. **a** L sequence, **b** M sequence, **c** S sequence and **d** partial Oya virus S sequence (365 bp). The *black triangle* indicates DHL10M107.

Phylogenetic analysis of the viral L and M gene sequences indicated that DHL10M107 clustered with MANV (VN04-2108) and an Oya virus (OYV) strain (SC0806) isolated from mosquitos in Sichuan province, China [17], although it was more closely related to VN04-2108 (Figure 1a, b). However, in terms of the S fragments, while DHL10M107 was in the same clade as VN04-2108 and SC0806, it formed a secondary clade, and the S fragments of VN04-2108 and SC0806 were more closely related (Figure 1c). Regardless of the genome fragment, DHL10M107 was closely related to three MANV strains (TRVL3587, SA An 4165, and AV 782; Figure 1). The OYV reported by Kono et al. [18] had a partial sequence for the S fragment in GenBank (accession no. AB075611). Similar to Figure 1c, the phylogenetic tree constructed using this sequence indicated AB075611, DHL10M107, VN04-2108, and SC0806 were in the same clade and had similar evolutionary relationships (Figure 1d).

We then compared the homology of the open reading frame (ORF) and amino acid sequences in DHL10M107 to five other *Orthobunyavirus* strains (Table 1). There was minimal nucleotide and amino acid homology between DHL10M107 and Tahyna virus, BATV, and Oropouche virus (Table 1). In terms of nucleotide homology, the similarity of the DHL10M107 L fragment to VN04-2108 and SC0806 was 96.3 and 92.6%, respectively. The M fragment (VN04-2108: 97.7% and SC0806: 97.2%) and S fragment (VN04-2108: 98.7% and SC0806: 98.9%) had similarly high levels of homology. Amino acid homology was between 99 and 100% for DHL10M107, VN04-2108, and SC0806 (Table 1). All of the amino acid differences were in the L and M fragments. In the L fragment, DHL10M107 differed from VN04-2108 by six amino acids and SC0806 by 11 amino acids. In the M fragment the difference was four (VN04-2108) and five amino acids (SC0806; Table 2).

**Table 1:** Nucleotide and amino acid homology in the sequences from DHL10M107 and other Orthobunya viruses

Genome segment and strain		% Nucleotide and amino acid sequence identities					
		1	2	3	4	5	6
Small							
1 DHL10M107			*98.7*	*98.9*	54.2	51.4	72.2
2 Manzanilla virus/VN04-2108		*100*		*99.6*	54.8	50.8	72
3 Oya virus/SC0806		*100*	*100*		54.9	51.1	72.2
4 Tahyna virus/XJ0708		43	43	43		54.4	52.8
5 Batai virus/MM2222		39.1	39.1	39.1	43.1		51.1
6 Oropouche virus/TRVL-9760		74	74	74	44.3	43.4	
Medium							
1 DHL10M107			*97.7*	*97.2*	46.2	48.9	59
2 Manzanilla virus/VN04-2108		*99.2*		*97.5*	46.1	48.9	58.9
3 Oya virus/SC0806		*99.3*	*99.2*		46.2	49	58.9
4 Tahyna virus/XJ0708		31.5	31.5	31.4		51.2	45.4
5 Batai virus/MM2222		32.7	32.8	32.8	43		48.5
6 Oropouche virus/TRVL-9760		52	52	52	30.5	31.3	
Large							
1 DHL10M107			*96.3*	*92.6*	55.9	56.1	65
2 Manzanilla virus/VN04-2108		*99.5*		*93*	56	56.2	65.1
3 Oya virus/SC0806		*99*	*99.2*		55.9	55.8	64.9
4 Tahyna virus/XJ0708		50.8	50.9	51.2		58.9	56.2
5 Batai virus/MM2222		48	48.2	48.3	54.2		55.3
6 Oropouche virus/TRVL-9760		31.7	32.2	31.7	23.8	23.3	

The percent nucleotide sequence identities are in the upper right half of the matrix and the percent amino acid sequence identities are presented in the lower left half.

Virus strains in italics (homologous values) are members of Manzanilla virus complex of genus *Orthobunyavirus*.

**Table 2:** Sites of amino acid differences in L and M sequences of DHL10M107, VN04-2108 and SC0806

Genome segment and strain	Sites of amino acid												
Large	150	274	289	435	456	467	484	487	922	1,202	1,634	1,660	2,055
DHL10M107	H	E	N	D	T	C	N	T	K	T	E	G	V
VN04-2108	H	E	N	D	N	R	S	T	N	T	D	E	V
SC0806	Q	G	E	E	T	R	S	G	K	V	D	E	A
Medium	20	349	370	395	714	725	1,139	1,222					
DHL10M107	T	D	T	Y	S	N	T	A					
VN04-2108	A	D	T	H	S	S	T	T					
SC0806	T	E	I	H	P	N	A	A					

*H* histidine, *E* glutamate, *N* asparagine, *D* aspartate, *Q* glutamine, *G* glycine, *T* threonine, *C* cysteine, *R* arginine, *S* serine, *K* lysine, *V* valine, *A* alanine, *I* isoleucine, *Y* tyrosine, *P* proline.

In conclusion, the high degree of similarity in the whole genome sequence between DHL10M107 and the four MANV strains in GenBank, but not other viruses in the Simbu serogroup, confirmed it was a MANV strain. This is the first report of MANV in mainland China. Currently, the records pertaining to MANV in the International Committee on Taxonomy of Virus (ICTV) [19] excludes Oya virus. Here, we identified two virus strains named OYV in GenBank. One was a partial OYV S fragment sequence (AB075611) isolated from the lungs of pigs thought to have Nipah virus infection in Malaysia [8]. The second strain was the entire sequence of OYV SC0806 (JX983192, JX983193 and JX983194) isolated from mosquitos in Sichuan province, China [7]. Phylogenetic analysis indicated that both OYV sequences clustered with the MANV strains. They were most closely related to VN04-2108 and DHL10M107, suggesting that OYV (SC0806 and AB075611) was also a member of the MANV complex.

## Additional files

Additional file 1:
**Table s1.** Specific primers for Manzanilla virus used in this study.

## Abbreviations

L: large RNA genome fragment
M: medium RNA genome fragment
S: small RNA genome fragment
MANV: Manzanilla virus
BATV: Batai virus
BHK-21: baby hamster kidney cells-21
CPE: cytopathic effects
OYV: Oya virus
ORF: open reading frame
ICTV: International Committee on Taxonomy of Virus
