# Supplementary material for: Isolation and full-length genome analysis of mosquito-borne Manzanilla virus from Yunnan Province, China
Source: BMC Res Notes. 2015 Jun 23;8:255. doi: 10.1186/s13104-015-1198-5 (PMC4477421; doi:10.1186/s13104-015-1198-5)
Supplement: Additional file 1: — Table s1. Specific primers for Manzanilla virus used in this study. [file 13104_2015_1198_MOESM1_ESM.docx]

**Additional file 1. Specific primers for Manzanilla virus used in this study.**

| Genome segment | Primer | Sequence (5'→3') |
| --- | --- | --- |
|
| Small |  |  |
|  | DHL107_S_1F | ATGGCAGATGCGATAGTTTTCAAT |
|  | DHL107_S_679R | TTAGATACGGATTCCAAATTGAGC |
|  | DHL107_S_504R | AAGTTGACATCCATCCCATCTTTC |
|  | DHL107_S_302F | CGAGATGGCTGATGACTTTCTATG |
|  | DHL107_S_OYA_1F | AGTAGTGTACTCCACAATTCAAAA |
|  | DHL107_S_OYA_966R | AGTAGTGTGCTCCCAATTCAAAGA |
|  | DHL107_S_OYA_504F | GAAAGATGGGATGGATGTCAACTT |
| Medium |  |  |
|  | DHL107_M_1F | ATGATCGCTGTACTTTTGCTATCA |
|  | DHL107_M_1025R | AATCATCTGCTAACTCGTCTAACT |
|  | DHL107_M_898F | TACAAGTCATTGAGCAAAGCCAGG |
|  | DHL107_M_2015R | TATCTAGCTCCGAGTCTGAATTCC |
|  | DHL107_M_556R | TTGAAATACCTCACACATGACCT |
|  | DHL107_M_433F | ACTACTGTGATAAATGGCTGGTT |
|  | DHL107_M_1337R | GCATGACTCCTGTTGCTTGTGCA |
|  | DHL107_M_1244F | ACTACTCTGTGACAAATAGGTGC |
|  | DHL107_M_OYA_226F | ACCAAGAAAAGAAGGCAAGGT |
|  | DHL107_M_OYA_775R | TAGCCGGGTAAAAGACAGGGA |
|  | DHL107_M_2759F | TCAGTTGTAAAAGAAGACCTCGA |
|  | DHL107_M_1-1F | AGTAGTGTACTACCACATACAACA |
|  | DHL107_M_749F | TTAATGATCCCTGTCTTTTACCCG |
|  | DHL107_M_1772R | AGAGTTGCCTTTCATAGTGTTGAG |
|  | DHL107_M_2250F | CAGACTTCAGTATCCACAATGAGG |
|  | DHL107_M_3211R | AATTCTTCACAGCCCCAATTACTT |
|  | DHL107_M_4025F | CGGTCAGGAAAATACAGCTTAACT |
|  | DHL107_M_4458R | AGTAGTGTGCTACCACGTACAAAC |
| Large |  |  |
|  | DHL107_L_1F | AGTAGTGTACCCCTAGGTTACAAC |
|  | DHL107_L_2059R | TTGTATAGGGAGCAAACTTTTCGG |
|  | DHL107_L_2325F | CATCACGTCCTAGTTGATCTC |
|  | DHL107_L_3527R | GGCACACTCTCTCATCACATCT |
|  | DHL107_L_4731F | ACCCACGATGATATTGAAACAGTT |
|  | DHL107_L_6905R | AGTAGTGTGCCCCTAGGAACATTA |
|  | DHL107_L_6510F | CATGGATCCGGAATTTCACCA |
|  | DHL107_L_6315R | AAGCAGTCCTAAATGCCCCT |
|  | DHL107_L_2324R | TGCGAGATCAACTAGGACGTGATGT |
|  | DHL107_L_2020R | ACATGACTAGACATTGCTAACGAGT |
|  | DHL107_L_2238R | GATCAACTTTACCAGGAAACCA |
|  | DHL107_L_5828R | ACCTATTCCTACCCCGTTGT |
|  | DHL107_L_1-1F | ATGAATCCTGATAAAATTGAGGAA |
|  | DHL107_L_3129R | CCACCCCATGCTCCTATCTT |
|  | DHL107_L_1092F | GGAAAGTTGATGGATTTCAGTGG |
|  | DHL107_L_1224R | TTAAACTGCTGCTCCCATAATGC |
|  | DHL107_L_OYA_3462F | TGGCTCCAAGGAAACATGAA |
|  | DHL107_L_OYA_4264R | ACCCATTCCATCATCAGTGGT |
